# Phenotypic and molecular characterization of the largest worldwide cluster of hereditary angioedema type 1

**DOI:** 10.1371/journal.pone.0311316

**Published:** 2024-12-26

**Authors:** Juan Sebastian Arias-Flórez, Sandra Ximena Ramirez, Bibiana Bayona-Gomez, Lina Castro-Castillo, Valeria Correa-Martinez, Yasmín Sanchez-Gomez, William Usaquén-Martínez, Lilian Andrea Casas-Vargas, Carlos Eduardo Olmos Olmos, Nora Contreras Bravo, Camilo Andres Velandia-Piedrahita, Adrien Morel, Rodrigo Cabrera-Perez, Natalia Santiago-Tovar, Cristian Camilo Gaviria-Sabogal, Ingrid Tatyana Bernal, Dora Janeth Fonseca-Mendoza, Carlos M. Restrepo

**Affiliations:** 1 Department of Morphology, Institute of Human Genetics, Grupo Investigación Genética Clínica UNAL, Universidad Nacional de Colombia, Bogotá D.C., Colombia; 2 Department of Internal Medicine, Hospital Universitario Mayor-Mederi, Universidad del Rosario, Bogotá D.C, Colombia; 3 Department of Pediatrics, La Cardio, and Universidad del Rosario, Bogotá D.C, Colombia; 4 School of Medicine, Universidad Nacional de Colombia, Bogotá D.C., Colombia; 5 Universidad de Boyacá, Medisens IPS, Tunja, Colombia; 6 Grupo de Genética de Poblaciones e Identificación, Institute of Human Genetics, Universidad Nacional de Colombia, Bogotá D.C., Colombia; 7 School of Medicine and Health Sciences, Universidad del Rosario, Bogotá D.C., Colombia; 8 School of Medicine and Health Sciences, Center for Research in Genetics and Genomics (CIGGUR), Institute of Translational Medicine (IMT), Universidad del Rosario, Bogotá D.C., Colombia; 9 Universidad Nacional de Colombia, Bogotá, Colombia; Universita degli Studi di Roma Tor Vergata, ITALY

## Abstract

Hereditary angioedema type 1 (HAE1) is a rare, genetically heterogeneous, and autosomal dominant disease. It is a highly variable, insidious, and potentially life-threatening condition, characterized by sudden local, often asymmetric, and episodic subcutaneous and submucosal swelling, caused by pathogenic molecular variants in the *SERPING1* gene, which codes for C1-Inhibitor protein. This study performed the phenotypic and molecular characterization of a HAE1 cluster that includes the largest number of affected worldwide. A geographically HAE1 cluster was found in the northeast Colombian department of Boyaca, which accounts for four unrelated families, with 79 suspected to be affected members. Next-Generation Sequencing (NGS) was performed in 2 out of 4 families (Family 1 and Family 4), identifying the variants c.1420C>T and c.1238T>G, respectively. The latter corresponds to a novel mutation. For Families 2 and 3, the c.1417G>A variant was confirmed by Sanger sequencing. This variant had been previously reported to the patient prior to the beginning of this study. Using deep-learning methods, the structure of the C1-Inhibitor protein, p.Gln474* and p.Met413Arg was predicted, and we propose the molecular mechanism related to the etiology of the disease. Using Sanger sequencing, family segregation analysis was performed on 44 individuals belonging to the families analyzed. The identification of this cluster and its molecular analysis will allow the timely identification of new cases and the establishment of adequate treatment strategies. Our results establish the importance of performing population genetic studies in a multi-cluster region for genetic diseases.

## Introduction

Hereditary angioedema HAE; OMIM #106100 is rare highly variable, insidious, and potentially life-threatening autosomal dominant condition, characterized by sudden local, often asymmetric, and episodic subcutaneous and submucosal swelling [1].

A global estimated prevalence rate of HAE ranges from one case between 50.000 to 100.000 inhabitants. There are no identified differences based on gender [2].

The pathogenesis of HAE is complex and involves mechanisms mediated by C1 and other serine protease inhibitors, resulting in excessive production of bradykinin which activates inflammatory phenomena followed by dilation, edema, and changes in vascular permeability. Edema attacks in HAE are nonpruritic and self-limiting with poorly defined margins and can affect different anatomical locations (mainly face, extremities, genitals, respiratory tract, and intestinal or mesenteric structures) [3]. Laryngeal edema usually occurs after oral trauma, such as dental surgery, but can also occur spontaneously [4].

The *SERPING1* gene (OMIM *606860) encodes the protein C1-Inhibitor protein (serpin peptidase inhibitor or complement factor C1 inhibitor), a key regulator in the complement system. Heterozygous pathogenic molecular variants in this gene can lead to HAE1, which accounts for ~85% of cases and is characterized by reduced synthesis of C1-Inhibitor protein. Alternatively, these variants may result in hereditary angioedema type 2 (HAE2), where C1-Inhibitor protein is synthesized at normal levels, but its function is impaired, and corresponds to ~15% of cases. It has been estimated that 5.6 to 25% of molecular variants related to HAE1 and HAE2 are the novo, making the *SERPING1* gene a prime example of mutagenic lability [5, 6] Other forms of hereditary angioedema with normal-C1-Inhibitor protein levels such as HAE3 (OMIM #610618), HAE4 (OMIM #619360), HAE5 (OMIM #619361), HAE6 (OMIM #619363), HAE7 (OMIM #619366) and HAE8 (OMIM #619367), are caused by pathogenic molecular variants in the *F12*, *PLG*, *ANGPT1*, *KNG1*, *MYOF* and *HS3ST6* genes, respectively [3]. These less frequent subtypes involve different pathophysiological mechanisms that lead to increased vascular permeability, with subtle clinical differences related to age of onset, sex predominance (primarily in females), disease triggers (mainly exposure to high estrogen levels and pregnancy), and the presence or absence of erythema marginatum or urticaria. However, these particularities are inconsistent across cases, making clear clinical differentiation difficult [7].

Due to the intricate nature of HAE and its associated risks, patients need to receive a precise diagnosis early in life; however, the delay in diagnosis is a prevalent issue among HAE patients globally. In Latin America HAE continues to be a highly under-recognized and under-treated disease; prevalence data suggest that at least 11.000 individuals are affected in the region. In fact, 575 cases have been reported across 10 Latin American countries, representing 5.2% of the expected cases [8]. Nowadays, data from Colombia include 44 patients with an average age at a diagnosis of 37 years, 95% of them had a diagnosis of HAE1 and 5% HAE2. Positive family history for the disease was documented for 88.23% and 62% are currently receiving treatment [9]. Sanchez et al, 2015, analyzed the impact of the disease on the quality of life in Colombian families using the 36-Item Short Form Survey (SF-36) and the 27-item quality of life questionnaire (QOL) KIDSCREEN-27 they evaluated the quality of life, psychological, well-being, and emotional performance indicators and found all of them significantly affected to whom suffered the disease [10].

In the present study, we conducted clinical, molecular and population characterization of a cluster with at least four families affected by HAE1, located in a rural region of Colombia. Our analysis, using whole exome sequencing (WES) allowed us to identify two molecular *SERPING1* gene variants (including one novel pathogenic molecular variant that has not been previously reported). Another variant previously reported in the patient was analyzed by Sanger sequencing (SS). The genotype-phenotype correlation was studied. Using deep-learning analysis, we established the structural modification of the C1-Inhibitor protein generated by *SERPING1* c.1420C>T, p.Gln474* and c.1238T>G, p.Met413Arg molecular variants, thereby contributing to the understanding of the molecular etiology of the disease. In addition, and to our knowledge, we describe the largest cluster of HAE1 reported worldwide.

## Materials and methods

### Sampling and data collection

The four families studied are originally from the Boyacá department, located in the Andean mountains in the central-eastern region of the country. Patients and their families were recruited during the period from May 7, 2022, to February 24, 2024.

**Fig 1:**
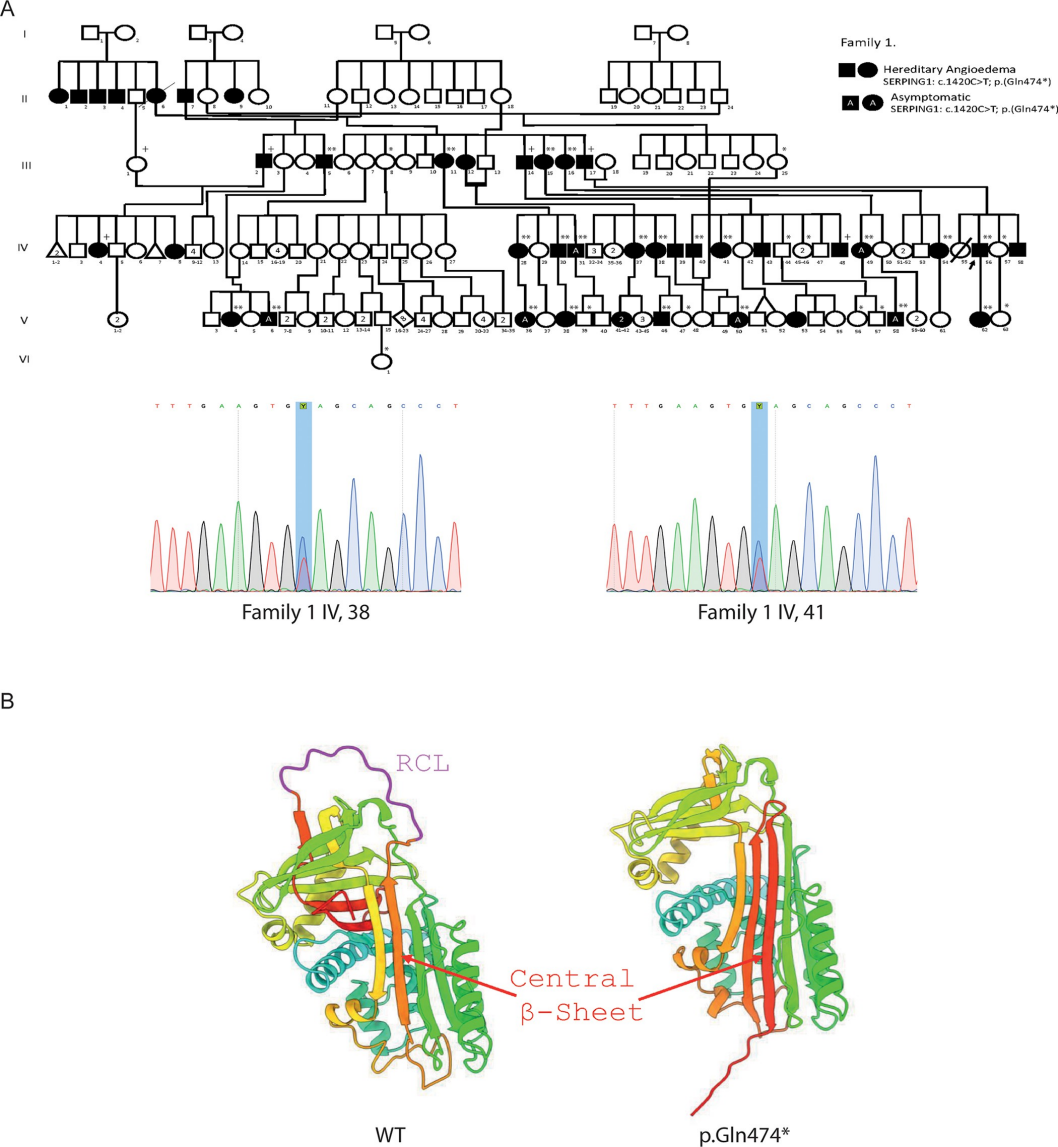
SERPING1 variant on Family 1 A) Pedigree of Family 1 with persons harboring the variant c.1420C>T p.(Gln474*) and Sanger sequencing confirmation of IV,38 and IV,41.+WES and *or ** negative or positive SS respectively. B) Left, the wild-type SERPING1 protein structure with the reactive center loop (RCL) highlighted in magenta. The central beta-sheet region is indicated in red; right, SERPING1 protein structure featuring the Gln474* variant. The molecular variant promotes the insertion of the RCL into the central beta-sheet, mimicking the latent form of the protein. The absence of the RCL region is shown, and the central beta-sheet is indicated in red. The overall conformational change depicts the predicted structural transition associated with the pathogenic variant.

Family 1 is originally from the countryside around the municipality of Toca, comprising 179 members. Of these, 42 were suspected to be affected with HAE1, and 27 were clinically evaluated. A total of 40 blood samples were obtained from this family for molecular analysis (33 by SS and 7 by WES) (Fig 1A).

**Fig 2:**
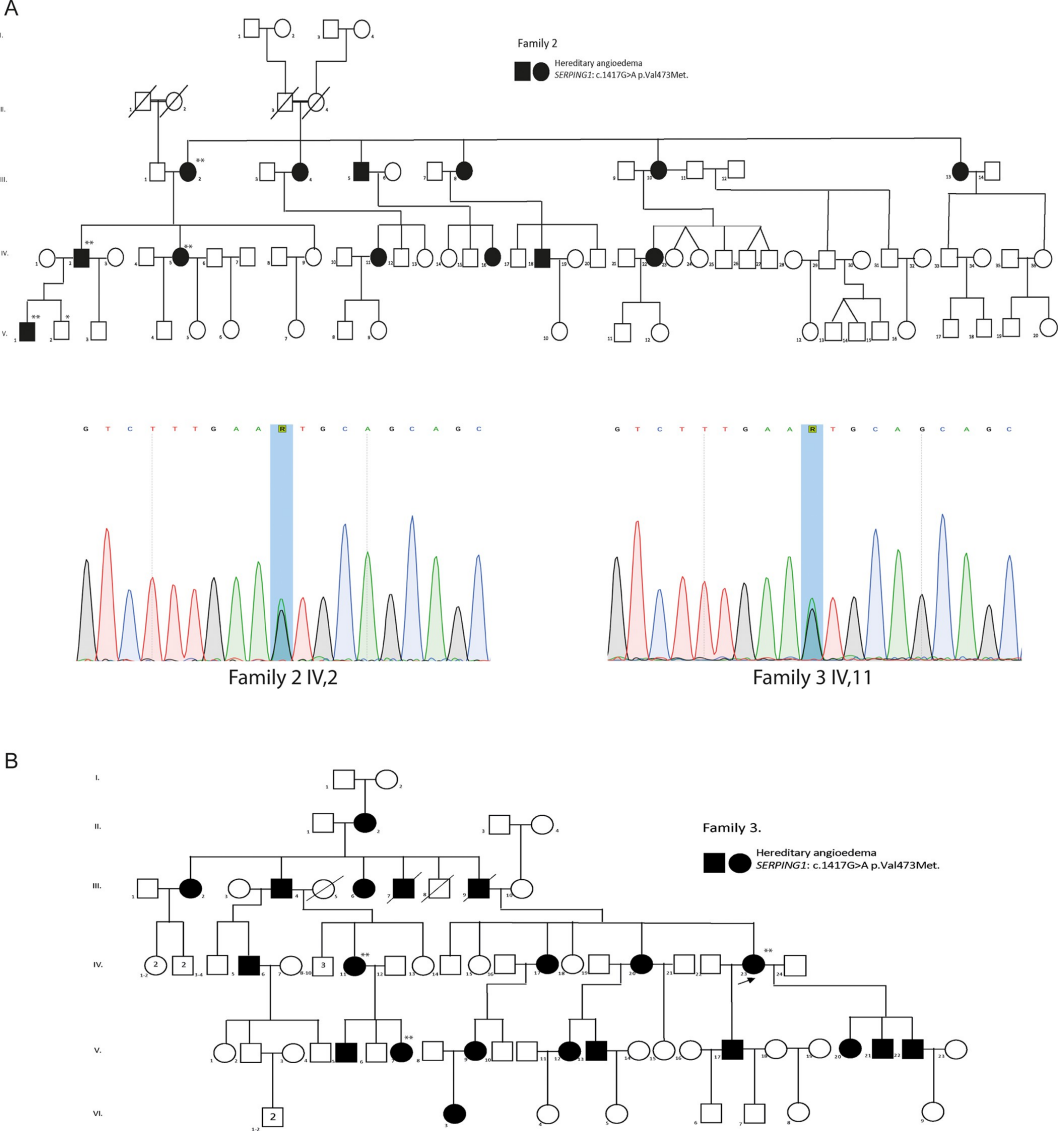
SERPING1 variants on Family 2 and 3. A) and B) Pedigrees of Family 2 and with persons harboring the variant c.1417G>A p.Val473Met and the Sanger Sequencing confirmation of Family 2 IV,2 and Family 3 IV,11. *or ** negative or positive SS respectively.

Family 2 is from the municipality of San José de Pare, comprising 78 members, with 13 suspected to be affected by HAE1. Four of these were clinically evaluated, and molecular analysis was indicated by SS. Additionally, blood samples were collected from 2 unaffected family members (Fig 2A).

Family 3 is also from San José de Pare and consists of 72 individuals, 21 of whom are suspected to be affected. Three were clinically evaluated and analyzed molecularly by SS (blood samples were obtained) (Fig 2B). No member of Families 2 and 3 was analyzed by WES by us, as an external institution had already identified the causal variant.

**Fig 3:**
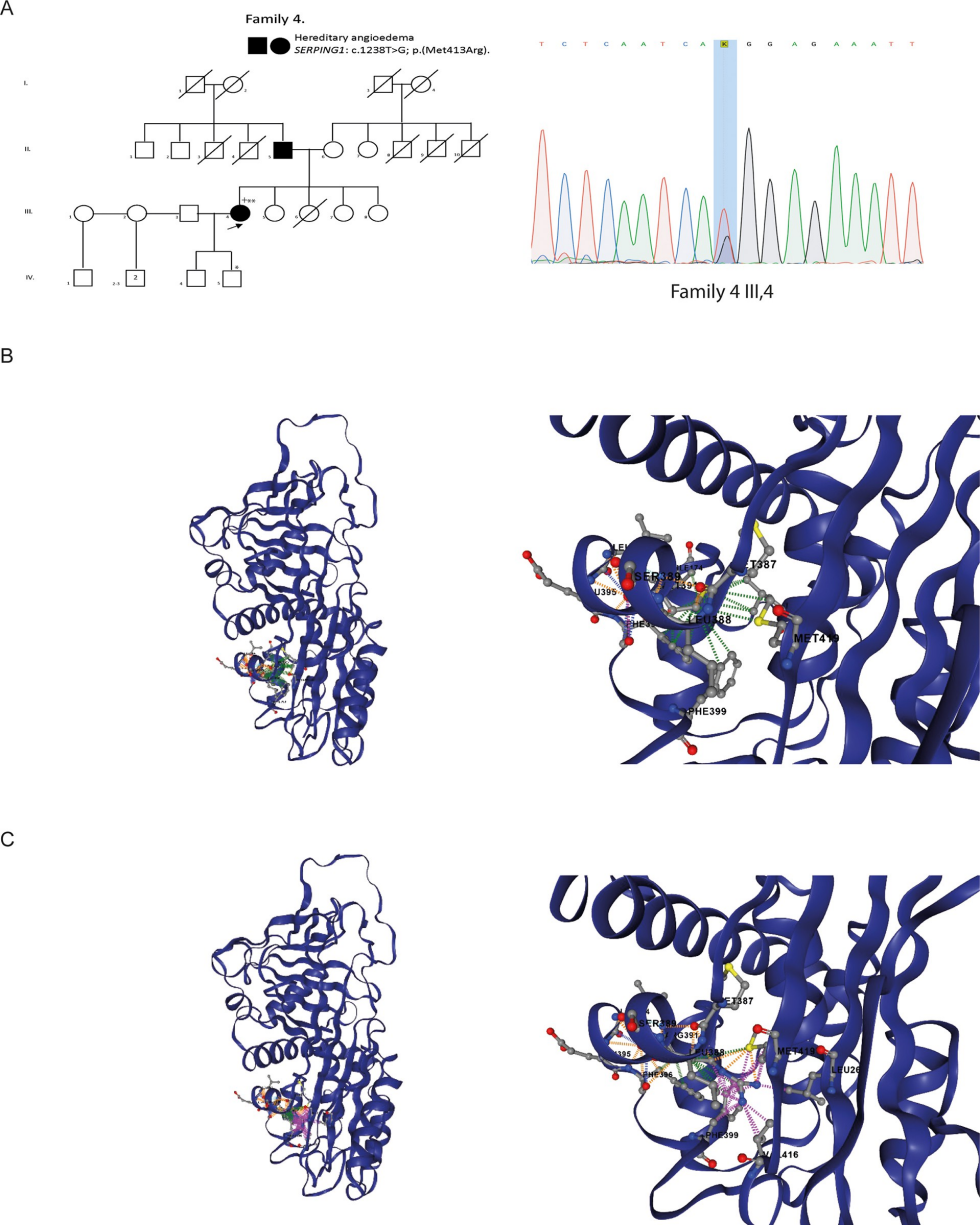
SERPING1 variants on Family 4 A) Pedigree of Family 4 with persons harboring the variant c.1238T>G p.(Met413Arg) and the Sanger Sequencing confirmation of the Family 4 III,4..+WES and *or ** negative or positive SS respectively B) and C) DynaMut2 protein stability change predictions (ΔΔGstability) were determined by uploading the experimentally derived structure of C1-inhibitor protein to the DynaMut2 server at https://biosig.lab.uq.edu.au/dynamut2/. Dashed lines predict intramolecular interactions. Predicted hydrophobic interactions are shown in green, intramolecular clashes in pink, hydrogen bonds in orange, and carbonyl interactions in blue. (A-B) WT C1-inhibitor protein, (C-D) Met413Arg C1-inhibitor protein.

Finally, Family 4 is originally from Tunja, the capital of the Boyacá department, and is composed of 27 individuals, 3 of whom have been clinically diagnosed with HAE1. A blood sample was obtained from 1 affected individual, who was analyzed by WES and SS. Additionally, SS was performed on her son, who was clinically suspected of having the disease (Fig 3A).

In total, across the four families analyzed, 79 individuals were suspected of having hereditary angioedema (HAE1), primarily based on physical examination and family history. Quantitative complement factor analysis was available for only 15 individuals.

Patients underwent assessment, including clinical history and physical examination conducted by geneticists. Extensive pedigrees were drawn, encompassing all members of the four families, and medical photographs were taken surveyed for clinical, socio-demographic and laboratory data. The patients and their relatives who agreed to participate in the study signed an informed consent/assent form after receiving a detailed explanation of the study.

All experimental procedures in this study were approved by the Ethics Committee of Universidad del Rosario (Approval DVO005-1614-CV1441, June 2021) and followed the Helsinki Declaration principles. The informed consent/assent was obtained from the study participants before the commencement of research activities.

### Whole-Exome Sequencing (WES)

Whole exome sequencing (WES) analysis was conducted on 8 patients of whom 1 belonged to Family 4 and 7 to Family 1 (including 2 asymptomatic controls). For WES Total genomic DNA was obtained from blood samples using the Quick-DNA Miniprep Plus Kit (Zymo Research). The library preparation and next-generation sequencing were performed by GencellPharma (Bogota, Colombia). The quality and quantity of DNA were assessed by using the Quantifluor ONE dsDNA system on a GloMax Discover instrument (Promega). Library preparation was conducted utilizing 250 ng of DNA using the MGIEasy FS DNA Library Prep Kit. Enzymatic DNA fragmentation was performed to yield fragments ranging from 200 to 400 bp, followed by end repair and PCR amplification. The Exome Capture V5 probe and streptavidin beads were used to capture the specific regions of interest. For enrichment, specific primers were designed in a final PCR reaction. For sequencing, the DNA was circularized, and the library underwent denaturation following split oligo ligation, followed by digestion and purification using specific beads. The circularized DNA was utilized to produce DNBs (nanoballs) via the rolling circle amplification process (MGI Tech Co, 2022). Subsequently, DNBs were quantified and subsequently sequenced on the DNBSeqG400 platform. The obtained reads were mapped to the hg19 reference genome employing the Burrows-Wheeler Aligner (BWA) and organized using SAMtools (https://github.com/samtools/samtools). Duplicate reads were detected and eliminated using Picard software to generate the BAM files (https://broadinstitute.github.io/picard/). Coverage and depth analysis were performed using the BAMBA tool, with a threshold of 50X considered acceptable. >93% of total bases called had a Phred-scaled quality score greater than 30 (>Q30). Finally, the vcf files were generated through the Haplotyper algorithm (Sentieon) and analyzed using VarSeq v2.3.0 software (Golden Helix). The bioinformatics analysis was conducted following the guidelines established for the classification of pathogenicity as described by The American College of Medical Genetics (ACMG) guidelines [11].

### Sanger sequencing

Genomic regions flanking the *SERPING1 (*NM_000062.3) gene containing the variants of interest were amplified by polymerase chain reaction (PCR). The primers amplification sequences for *SERPING1*_c.1417G>A and c.1420C>T were F: 5´cacagcatcatggcattgcat3´ and R: 5´cagggagcccttttggtggatag3´. For *SERPING1* c.1238T>G were F: 5´ agcccttctgttttcaaggccat 3´and R: 5´ ggtgttctggtttgcctctgact 3´. The PCR conditions were as follows: initial denaturation at 95°C for 10 min; 35 cycles consisting of 95°C for 40s, 60°C for 40s, and 72°C for 40 s; and final extension at 72°C for 10 min. The PCR products were visualized on agarose gels (1.5%) by ethidium bromide staining. The PCR product length was 735pb for c.1417G>A/ c.1420 C>T and 380pb for c.1238T>G. Subsequently, The PCR-amplified products were purified by alkaline phosphatase and exonuclease I and directly sequenced. The reference sequence was obtained from Ensembl (ENST00000278407.9). Sequences were analyzed using FinchTV v.1.5.0 (Geospiza Inc) and aligned with reference sequences by Clustal W v2.1 ((http://www.clustal.org/). SS was performed on 33 individuals from Family 1, six from Family 2, three from Family 3, and two from Family 4, as described in the Samples and Data Collection section (Figs 1A, 2A, and 3A).

Protein structure prediction for *SERPING1* p.Gln474* and p.Met413Arg variants.

The three-dimensional structures of *SERPING1* variant (NM_000062.3) for both the wild type (WT) and Gln474* variant, were predicted using AlphaFold2, a deep learning-based protein structure prediction tool [12]. For this analysis, the sequences were processed using the AlphaFold2 source code at the GitHub repository (https://github.com/google-deepmind/alphafold) using the ‘—db_preset = reduced_dbs’ parameters to streamline the computational effort and using ‘—max_template_date = 2022-01-01’. The prediction was confirmed by uploading the sequence to the trrosetta server [13]. Since Alphafold2 has limited ability to in model protein stability and dynamics, we used Alphamissense [14] and DynaMut2 [15] to predict the variant’s effects on protein stability and function, by using the experimentally determined 5DU3 pdb structure as a base structures were displayed using ChimeraX [16].

### Statistical analysis

Statistical differences between the clinical characteristics of the evaluated families were assessed using ANOVA analysis for the parametric variables and the Kruskall-Wallis test for the nonparametric variables. The *P* value for significance was set at < 0.05. We performed post hoc analysis using Tukey tests. The analyses were conducted using Jamovi v.2.3.28.0.

## Results

### Clinical findings in HAE1 patients

We examined a cohort comprising 35 individuals diagnosed with HAE1. Among these individuals, 29 were affected by the condition, with symptoms manifesting at an average age of 13.1 years (range: 2–45 years) and 6 were asymptomatic. The clinical and biochemical diagnosis was established within a wide age range, spanning from 2 to 65 years old (mean age 26.3 years). Gender distribution within the cohort was biased towards females constituting 58.3% of the participants (female-to-male ratio 3:2). On average, individuals experienced 14.9 edema crises annually. Two patients exhibited the highest frequency of episodes, experiencing 48 crises per year, while three patients had only one episode per year.

Regarding body location, edema predominantly affected the face and abdomen, with 23 patients experiencing swelling in each of these locations; edema with airway obstruction was described in 51.7% of affected individuals. Although some males referred to swelling in the testicular and perineal areas, such occurrences were infrequent. Orotracheal intubation secondary to severe neck and airway edema was recorded in 17.2% (5/29) of patients. Death associated with HAE1 occurred in 5 cases and the number of episodes tended to increase with age.

A total of 86.2% of patients received specific treatment for the disease, mainly with Icatibant and Lanadelumab. Icatibant was administered for acute episodes and Lanadelumab for long-term management. Among the participants, 16 individuals met the criteria for long-term treatment. However, 20.7% of those with HAE1 received Lanadelumab due to difficulties associated with medical prescription and delivery in geographically rural areas.

The occurrence of edema was reported to be both spontaneous and triggered by several factors. Among the triggers and their percentages, we have the following as pregnancy 18% (3/16), menstrual cycle 25% (4/16), stress 20.7% (6/29), physical activity 20.7% (6/29), minor traumas 31% (9/29), cold exposure 13.8% (4/29), fasting 10.3% (3/29), the nighttime 6.9% (2/29), alcohol 37.9% (11/29) and ingestion of various foods including eggs, fish and grains, fruits such as melon, watermelon, papaya and acidic flavors. Quantitative complement factors data was available for 15 individuals affected with HAE1. It is noteworthy that all measurements of patients showed decreased levels of C1 inhibitor (C1-Inhibitor protein) and C4, consistent with the diagnostic criteria for HAE1 (for more detailed data, refer to S1 Table).

The clinical characteristics and statistical analyses among the analyzed families are described in Table 1.

**Table 1:** Clinical characteristics of patients with HAE.

Clinical characteristics of patients with HAE				Family 1	Family 2	Family 3	Family 4	p value
			Mean (%)	Mean (%)	Mean (%)	Mean (%)	Mean (%)	
**n**			35	27	4	3	1	
**Age (years)**			35.1	33.4	38.5	40.7	49.0	0. 755
**Sex F**			21 (58.3)	15.0	2	3.0	1.0	
**Sex M**			14 (41.6)	12.0	2	0.0	0.0	
**Sex Ratio**		Male:Female	14:21	12:15	2:2	0:3	0:1	0.289
**Age of onset (years)**			13.1	10.95	20.3	20.3	7	**0.039**
**Age at diagnosis (years)**			26.3	24	20.3	20.3	21	0.242
**No. Attacks/year**			14.9	15.05	9	15	36	0.925
**Intubation requirement**			5 (17.2)	4 (19)	0	0	1 (100)	0.262
**Edema localization (n = 29)**		Face	23 (79.3)	18 (86)	2 (50)	2 (67)	1 (100)	0.581
		Airway	15 (51.7)	12 (57)	1 (25)	1 (33)	1 (100)	0.375
		Abdomen	23 (79.3)	18 (86)	2 (50)	3 (100)	0	0.279
		Skin	20 (69)	14 (67)	3 (75)	2 (67)	1 (100)	0.813
		Asymptomatic patients	6 (20.7)	6 (29)	0	0	0	
**Biochemistry**								
**C4**		C4 (RV: 10–40 mg/dl)	3.52	3.5	7.0	NI	NI	0.244
**C1-INH**	C1-INH-Ag (RV: 19–37 mg/dl)		5.525	5.7	3.8	6.0	NI	0.841
	% function (RV: 70–130%)		20%	0.2	0.2	0.5	0.2	0.212
**Treatment**								
**Receives treatment**		Yes	25 (86.2)	19 (90)	2 (50)	3 (100)	1 (100)	0.469
**Acute attack**		Icatibant	24 (82.8)	19 (90)	1 (25)	3 (100)	1 (100)	0.645
**Prophylaxis**	**Short-term**	Danazol	7 (24.1)	7 (33)	0	0	0	0.986
		Tranexamic acid	1 (3.4)	1 (33)	0	0	0	
	**Long-term**	Lanadelumab	6 (20.7)	5 (24)	0	0	1 (100)	0.733
		Berinert	1 (3.4)	1 (5)	0	0	0	

HAE: Hereditary Angioedema; RV: Reference Value; NI: No information; n: Number; F: Female; M: Male.

### Molecular characterization

WES analysis performed on 8 patients belonging to families 1 (5 patients and 2 control) and 4 (1 patient) allowed for the identification of two molecular variants in the *SERPING1* which corresponded to c.1420C>T, p.Gln474* and c.1238T>G, p.Met413Arg respectively. In Family 1, the mutation was found in all 5 affected patients, and in Family 4, the evaluated patient tested positive.

For Families 2 and 3, the missense variant *SERPING1* c. 1417G>A, p.Val473Met had been previously reported, prior to the beginning of this study.

The classification of the pathogenicity of the variants was performed following the ACMG guidelines [11]. Missense molecular variant c.1417G>A, p.Val473Met is considered pathogenic and fulfills criteria PS4, PM2, PM1, PM5, PP3, and PP5. Nonsense variant c.1420C>T, p. Gln474* is classified as pathogenic, meeting criteria PS4, PVS1, PM2, and PP5. Both variants are described as pathogenic in ClinVar and LOVD public databases. Finally, a missense change *SERPING1* c.1238T>G, p.Met413Arg, has not been reported in the literature and is considered novel. This molecular variant is classified as Likely pathogenic and meets criteria PM1, PM2, PP3 and PP4.

The Sanger sequencing analysis was performed on 44 individuals, comprising thirty-three from Family 1, six from Family 2, three from Family 3, and two from Family 4. In Family 1, the *SERPING1* c.1420C>T molecular variant was identified in 66.6% of those evaluated (22 out of 33). Of the cases with a positive molecular diagnosis, 27.3% were asymptomatic (6 out of 22) (Fig 1A). *SERPING1* c.1417G>A pathogenic molecular variant was detected in 66.6% of those evaluated (4 out of 6) (Fig 2A). In Family 3, the *SERPINGN1* c.1417G>A molecular variant was identified in 100% of those evaluated (3 out of 3) (Fig 2B). Finally, the novel c.1238T>G likely pathogenic molecular variant identify using WES in one individual from Family 4, was confirmed by Sanger sequencing (Fig 3A).

The molecular analyses performed in the 4 families by WES and SS, indicate that 35 patients carry a mutation in *SERPING1*, of which 6 were identified by WES and 30 by SS (S1 Table)

### Protein structure prediction

In our results, the structural analysis of wild-type (WT) C1-Inhibitor protein and the Gln474* mutant, conducted through AlphaFold predictions, provided significant insights into the conformational dynamics of C1-Inhibitor protein transitioning towards its latent form. The beta-sheet architecture, a defining structural feature of C1-Inhibitor protein in its native state, facilitates its inhibitory function through a precise arrangement that allows the reactive center loop (RCL) to be readily accessible for interaction with target proteases. The transition to the latent form involves a critical structural rearrangement where the RCL is inserted into this beta-sheet, thereby rendering the inhibitor inactive by essentially locking the structure in a conformation that prevents protease binding [17].

Remarkably, the AlphaFold predictions for the Gln474* mutant closely mimic this latent state configuration. The mutant’s beta-sheet architecture showed a conformation that aligns with the characteristic RCL insertion into the beta-sheet observed in the latent form of C1-Inhibitor protein. This change, induced by the Gln474* pathogenic molecular variant, suggests a direct mechanism by which the pathogenic molecular variant may predispose C1-Inhibitor protein to a latent-like state, thus potentially impairing its physiological function as a serine protease inhibitor. The same conformational change is observed when the protein structure is modelled using trRosetta (Fig 1B) This insight underscores the profound impact of the Gln474* pathogenic molecular variant on the structural integrity of C1-Inhibitor protein, aligning with the transition mechanism from the active to the latent form of the protein.

On the other hand, the Met413Arg variant shows an Alphamissense score of 0.71, which predicts a pathogenic effect [14], as well as a reduction of hydrophobic interactions and simultaneous increase in intramolecular clashes, resulting in a predicted destabilizing change (ΔΔGstability) of -1.8 kcal/mol when using DynaMut2 (Fig 3B and 3C).

## Discussion

Hereditary angioedema (HAE), as a rare, chronic and debilitating disorder characterized by recurrent, unpredictable, and potentially life-threatening episodes of swelling. Despite this, due to its low prevalence and symptoms that mimic those of more common diseases, it is often misdiagnosed or inadequately diagnosed, leading to patients receiving late and ineffective treatment [3]. It is estimated that of the expected 11,000 HAE patients in Latin America, only about 5.0% have been reported [8]. In this context, our study significantly contributes to the understanding of this condition in countries with significant underdiagnosis.

The patients identified in our study belong to Boyacá, a department located in central-eastern Colombia, in the Andean region. This department has 1.3 million inhabitants (Colombian National Statistics System-DANE, 2023) [18], and according to our findings, we can estimate a prevalence of 1:16,883 affected with HAE in this area. This data is highly relevant as it significantly exceeds the estimated worldwide prevalence of 1:50,000–600,000 [19]. Additionally, according to the estimated HAE incidence for our country, which corresponds to 1:80,085, the impact of this genetic condition in this specific region of the country is evident [9].

Our finding of a higher number of cases than expected for HAE1 and the observation that our affected individuals live in a common area allows for the establishment, for the first time worldwide, of the presence of a HAE1 cluster, consisting of 79 suspected to be affected individuals located in the municipalities of Toca, San José de Pare, and Tunja, Boyacá-Colombia. According to previous literature reports, we estimate that the identified HAE1 cluster can be understood as a biosocial phenomenon, with an origin related to a combination of several factors including a) biological factors determined by the autosomal dominant nature of the disease; b) geographical factors as these populations are located in rural areas with low migration rates, and c) cultural factors, such as high birth rates, family-based agricultural economies, and a tendency to form unions within the same geographical area or among relatives to maintain land ownership within the family across generations [20].

A recent study has described the presence of 122 clusters of rare diseases or congenital anomalies in South America, placing Colombia in second place, after Brazil, with the highest percentage of these clusters (10.7%), predominantly represented by autosomal dominant diseases (46.1%) [21]. Interestingly, the HAE1 cluster identified by us would correspond to the fourth described for the department of Boyacá or the Andean region, where previous studies have reported frequencies of affected individuals higher than expected for mucopolysaccharidosis type III (San Filippo syndrome), Ellis Van-Creveld syndrome, and mucopolysaccharidosis type IV (Morquio syndrome) [20, 22, 23]. Taken together, these results support the estimation that Boyacá is a multi-cluster region, characterized by having communities with more than one genetic disorder, which has been recognized currently only for the Antioquian population, in which 6 genetic disease clusters have been identified [21]. The study of genetic clusters is the fundamental objective of medical population genetics, which has a great impact at the community level, supporting, as in our case, the identification of the genetic causes of the disease and risk factors in communities that, like the one evaluated by us, are concentrated in remote areas from genetic reference centers [24, 25].

Regarding the phenotypic characterization of patients, our findings demonstrated that overall clinical manifestations correspond to those reported in previous literature on HAE patients [1]. Interestingly, 79.3% of our patients presented with abdominal involvement, which exceeds the percentage evidenced in other primarily Asian populations, such as Japanese and Chinese individuals, with values of 38% and 69.9%, respectively [26, 27]. Nevertheless, as previously described, gastrointestinal (GI) involvement, resulting in abdominal pain occurs in 43% to 93% of patients, with the possibility of involving the entire GI tract, leading to a series of GI symptoms during an acute attack. It is important to highlight that GI involvement secondary to HAE is commonly confused with other disorders such as appendicitis, cholecystitis, and pancreatitis, which may affect the accurate diagnosis of HAE [28, 29]. Similarly, another main symptom reported by our patients corresponds to facial edema, which was present in 79.3% of those affected [30]. This predominance of affectation can be attributed to a combination of factors including increased vascular permeability, sensitivity to bradykinin, higher exposure to triggering factors, and the localization of soft tissues in the face [1, 31].

Significantly, we identified a high percentage (42%-58%) of patients with erythema marginatum, a prodromal symptom usually rare in other analyzed populations [32, 33]. This cutaneous rash may be more prominent in some affected patients due to a combination of factors such as complement system activation, higher systemic inflammatory response, a higher percentage of specific triggering factors such as trauma and stress, and individual genetic differences predisposing to a prolonged immune system response [34].

Given that HAE1 is a potentially life-threatening disease, timely diagnosis represents the minimization of risk for affected individuals. However, the panorama in Colombia has shown that the average age of diagnosis is 37 years, which represents a significant delay compared to other cohort reports where diagnosis occurs between 1.4 to 8.5 years after the initial clinical manifestation [1]. In the sample analyzed, an average age of diagnosis of 26 years was documented, which, like the national panorama, has approximately a 14-year delay in identifying the disease. This reality, which also affects other Latin American populations, may result from factors such as lack of disease awareness, absence of specialized professionals (immunologists, allergists, and geneticists), dispersion of the population in rural areas, and difficulties in accessing treatments in specialized centers (https://haei.org/wp-content/uploads/2016/03/HAEi-Report-1-Artwork_LATAM).

It has been estimated that although some attacks are not related to an identifiable trigger, many are associated with various factors, which for our cohort corresponded to pregnancy (18%), stress (20.7%), trauma (31%), food (37.9%), and cold (13.8%). These and other factors have been widely recognized as triggers of edema in HAE1-affected patients [1]. Like our cohort, other reports in the literature have demonstrated the impact of foods such as cheese, fish, tomatoes and pineapples, which contain o release histamine. In this context, a histamine intolerance reaction is likely associated with the induction of angioedema [35].

The average age of symptom onset in our patients was 13.1 years, which is consistent with global and local literature describing HAE symptoms as usually mild or nonexistent during early childhood, with typical manifestations during the first or second decade of life [9, 31]. Other literature reports have indicated that 40% of HAE patients experience their first episode before the age of 5 and 75% before the age of 13, with puberty being associated with symptom onset in women and men [36, 37]. In our cohort, the estimated penetrance of HAE1 at 13 years was 92.2%, and at 30 years has increased to 98.7%.

The patients studied presented an average of 15 attacks per year (AY), which is consistent with global and Latin American literature that has determined an average annual occurrence of 20.2 (SD 16.9) attacks, with varying ranges in different populations, such as Brazilian (11.3 AY), Canadian (17.4 AY), and German (25.6 AY) populations. Despite guidelines recommending that all patients carry on-demand treatment (ODT) in case of an attack and that all attacks be treated as soon as possible to minimize potential progression and mortality, multicenter studies worldwide have revealed that only 86% of patients receive a prescription for ODT, and about 10% of attacks go untreated [38]. Our data are similar, as it was evidenced that an average of 82.8% of patients with acute attacks were treated with Icatibant, a bradykinin B2 receptor antagonist, which reduces B2R-mediated vascular permeability and the time to initial symptom improvement [39]. Our data suggest a better outlook for acute attack treatment for patients in our cohort compared to those in other areas of our country, where acute crisis management with Icatibant has been reported in only 38.9% of patients [9]. This therapeutic management in our patients is relevant and successfully overcomes problems derived from lack of access, supply, and logistics to maintain medications used in approved ODT therapies for HAE in adequate conditions (e.g., temperature). In other countries such as the United States, significant disparities in access to HAE medications in rural areas have been reported, leading to inequality in these populations compared to residents in urban locations [40]. The success in managing patients in crisis in the area of our study is potentially due to community education that has generated self-care strategies and treatment administration outside of a medical center, a model that has been used by other cohorts in HAE treatment [41].

In addition to ODT therapy, the HAE-affected community in the analyzed cluster uses prophylactic therapy with danazol (24.1%) or tranexamic acid (3.4%), which is a lower percentage than that established for the country (62%) [9]. Like other literature reports, danazol is frequently used as prophylaxis against anticipated triggers such as surgeries or invasive dental procedures. The occurrence of secondary adverse reactions due to the use of this androgen has not been overlooked, making it contraindicated during pregnancy or in pediatric populations [38].

Long-term treatment using lanadelumab and Berinert was documented in 20.7% and 3.4% of our patients, respectively. The use of lanadelumab has been considered a revolutionary approach not only due to its ease and periodicity of administration (single injection once every 2–4 weeks) but also due to its effectiveness and tolerability. This medication represents the most widely used not only in our patients but in the country as a whole (Integrated Social Protection Information System—SISPRO https://www.sispro.gov.co/).

Interestingly, we note that Colombia is one of the Latin American countries, along with Argentina, Brazil, and Mexico, that has managed to ensure the availability of treatments for acute, prophylactic, or long-term attacks, which impact the economic burden associated with HAE, given its significant effect on patients, the healthcare system, and society [42].

It is acknowledged that the various subtypes of hereditary angioedema (types 1 to 8) present with heterogeneous clinical manifestations without apparent phenotypic differences. Consequently, the evaluation of C1-Inhibitor protein levels is necessary for accurate classification. In this context, in the absence of such data, molecular diagnosis is recommended [7, 43]. Although the definitive diagnostic impression for our patients was HAE1, only 42.9% had C1-Inhibitor protein measurements. Given the comprehensive analysis capabilities of WES and its application in rare disease studies, we conducted an analysis of additional genes beyond *SERPING1* that have been identified as causal for HAE (*F12*, *PLG*, *ANGPT1*, *KNG1*, *MYOF*, *and HS3ST6*) [44]. WES analysis allowed us to identify two pathogenic molecular variants in the *SERPING1* gene (c.1420 C>T, p.Gln474*, rs1565174105; and c.1238T>G, p.Met413Arg (novel molecular variant).

The molecular spectrum of pathogenic molecular variants causing HAE has been poorly explored in the country, with only one national publication to date that studied 22 patients from 4 unrelated families in the southern region of Colombia. This report identified 12 patients carrying the pathogenic molecular variants c.1081C>T (p.Gln361*), c.1396C>G (p.Arg466Gly), c.1029+84G>A, and c.106_107del (p.Ser36Phefs*21) [45]. These pathogenic molecular variants had been reported in other patients worldwide and are different from those identified in our cohort from another geographical region of the country. This finding is not surprising given that over 700 variants in the *SERPING1* gene, including pathogenic single nucleotide variants, small insertions or deletions, large deletions, and duplications, have been described [46]. Mutational heterogeneity has been identified in different populations, with a predominance of missense and in-frame variants (36.8%), frameshift (28.9%), nonsense (14.5%), splicing (13.2%), and gross deletions/duplications (6.6%) [26].

Two out of the three variants analyzed in our patients have been previously reported. The *SERPING1 c*.1420C>T, p.Gln474* variant was the most commonly identified variant in 27 patients belonging to Family 1 (5 using WES and 22 by SS). It is a nonsense variant found exclusively in heterozygosity, previously described in Mediterranean populations [47, 48]. Our structural prediction analysis of the Gln474* variant presents a compelling narrative. The AlphaFold-derived models suggest that this particular pathogenic molecular variant promotes a transition toward the latent form of C1-Inhibitor protein notably through the insertion of the reactive center loop (RCL) into the central beta-sheet, a hallmark of the latent state. This finding represents one of the first pieces of structural evidence suggesting a genetic variant’s propensity to favor such a significant conformational shift. The implications of this transition extend beyond mere structural alteration, hinting at a potential mechanism through which the Gln474* variant could diminish C1-Inhibitor protein´s inhibitory functionality. By favoring the latent conformation, characterized by its inability to effectively engage with target proteases [49], this pathogenic molecular variant could critically undermine the protein’s physiological role, offering a structural basis to understand the variant’s impact on C1-Inhibitor protein´s biological function.

Furthermore, the observed low plasma levels of C1-Inhibitor protein patients with the Gln474* variant can be explained through three primary mechanisms induced by this pathogenic molecular variant: 1) Altered Secretion: The pathogenic molecular variant may impair C1-Inhibitor protein´s proper folding and secretion, leading to its retention and degradation within the endoplasmic reticulum, thus reducing its plasma levels. 2) Increased Clearance: The latent form of C1-Inhibitor protein might be more susceptible to recognition and clearance from the plasma, either through enhanced proteolytic degradation or increased uptake by cells, further diminishing its circulating levels. 3) Formation of Polymers or Aggregates: This variant could also promote the aggregation of C1-Inhibitor protein into polymers that are sequestered within cells or deposited in tissues, thereby not only lowering its availability in plasma but potentially contributing to tissue damage [50]. Each of these mechanisms highlights the profound impact of the Gln474* variant on the structural integrity of C1-Inhibitor protein, offering insights into the observed phenotypic consequences in affected individuals.

The modeling of the protein C1-Inhibitor protein_p.Gln474* and our functional hypothesis of latent molecule formation allows us to classify it as Class III according to classification proposed by Bos et al. This classification is based on the inhibitory capacity of the C1 inhibitor, determined by its ability to bind and trap a protease through adequate exposure to the reactive center loop (RCL) and provides a foundation for predicting the phenotype associated with different genetic variants and for developing more targeted therapeutic strategies [51].

The second most frequent variant, found in 7 patients belonging to Families 2 and 3, corresponded to a missense variant *SERPING1* c.1417G>A, p.Val473Met, which has only been described in European populations [47, 52]. Interestingly, this is the first time that this pathogenic molecular variant has been identified in a Latin American population. Functional validation of this variant conducted by Haslund et al. demonstrate a significant decrease in protein C1-Inhibitor protein concentration. Western blot analysis revealed a dominant-negative effect, leading to reduced secretion and subsequent intracellular accumulation of C1INH [52]. It has been suggested that this effect may be related to the formation of aggregates in the endoplasmic reticulum, which alters cellular homeostasis. Recent literature reports support the hypothesis that a large proportion of *SERPING1* missense variants cause the disease through dominant-negative mechanisms, which has been crucial in the development of new treatments for hereditary angioedema, including antisense oligonucleotide treatment and gene therapies [44, 53, 54]. The p.Val473Met pathogenic molecular variant is situated within loop sheet 1C/sheet 4B within the gate region. Its displacement during reactive center loop (RCL) insertion results in the loss of Val473, which plays a role in the mobility of the gate. This loss is expected to have significant consequences for the formation of the serpin-protease complex. Therefore, classifying p.Val473Met within Class II is appropriate [55].

The genotype-phenotype correlation between the variants *SERPING1* c.1417G>A (Class II) and *SERPING 1*c.1420C>T (Class III) demonstrated statistically significant differences only in the age of symptom onset (p: 0.039). Our findings align with existing literature that has reported an earlier onset of symptoms in patients carrying Class III pathogenic molecular variants. Indeed, a recent study by Loli-Ausejo et al. (2021) established, like our cohort, that the median age of symptom onset for such pathogenic molecular variants was 10 years, with an interquartile range (IQR) of 3.5–21.5 [55]. However, unlike our study, the authors demonstrated statistical differences between patients with Class II and Class III pathogenic molecular variants. The trend observed by us and other authors regarding the early onset of symptoms in patients with Class III pathogenic molecular variants suggests more severe phenotypes associated with this type. For other clinical manifestations, no significant differences have been found among patients of Classes 0, I, II, and III, highlighting the need for large cohort studies involving patients with different types of pathogenic molecular variants to accurately establish genotype-phenotype correlations [55].

Finally, we found a new variant c.1238T>G, p.Met413Arg classified as Likely Pathogenic, that had not been previously described in the literature or reported in the clinical databases ClinVar, dbSNP or LOVD (Leiden Open Variation Database).

This new pathogenic molecular variant is located in the serpin domain of the C1-Inhibitor (residues 113 to 478), which is essential for the regulatory function of the complement cascade and coagulation, by inhibiting key proteases in these pathways. Interestingly, it has been reported that 96.3% of SERPING1 missense pathogenic molecular variant are located in this domain [5].

At position 413, there are two other changes reported in the databases (gnomAD and dbSNP) which correspond to p.Met413Leu and p.Met413Thr (rs780690190 and rs1945481705 respectively). The frequency for these variants is very rare (0.000001859 and 0.000001239 respectively) and our variant is novel. These observations indicate that in this codon the missense variants are infrequent.

The alignment of the proteins between human (P055155), rat (Q6P734) and mouse (P97290) showed that this amino acid is conserved between these species, which shows the importance of this residue (https://www.uniprot.org/, data not shown). According to AlphaMissense prediction, the p.Met413Leu and p.Met413Thr changes are probably benign and ambiguous respectively, but in our case, the p.Met413Thr change is probably pathogenic. These effects are probably due to the characteristics/properties of each amino acids.

Methionine is a hydrophobic AA and arginine is an AA with a positive charge. Due to its sulfur group, methionine has the property to stabilize the protein structure [56]. The Arginine, due to its atom composition, its charge, its flexibility and hydrophobicity properties, is a residue that promotes disorder [57]. The methionine residue is located in an alpha helix as shown in Fig 3B. The change could modify the stability of the protein structure as DynaMut2 predicts with a destabilizing change (ΔΔGstability) of -1.8 kcal/mol and could alter its function (Fig 3C).

Our study presents the clinical and genetic analysis of a cluster of patients affected by HAE1, comprised of the largest number of patients reported in a single geographical area. Our study contributes to the understanding of the disease in regions with underdiagnosis and poor molecular characterization, which impacts the ability to offer timely diagnosis and treatment.

### Study limitations

The present study has some limitations that should be noted. Firstly, segregation analysis could not be performed in family 4, which harbors a likely pathogenic new variant, thus hindering the identification of the molecular cause of the disease in this group. Secondly, some phenotypic data (e.g., C1-Inhibitor protein values in all members of families 1, 2, and 3) could not be obtained, impeding the establishment of total genotype-phenotype relationships. Finally, we deem it important to conduct functional validation analyses of the new variant to establish its impact on the etiology of the disease.

## Conclusion

Hereditary edema is a chronic condition that appears in most cases in adolescence. Our study describes and identifies such a large population with hereditary edema and allows us to obtain more information about the course of the disease, the impact on quality of life and the relationship with inbreeding. We were able to identify genetic and immunological markers, which will be used in treatment and thus improve the quality of life of patients. It is important to highlight that quickly identifying the symptoms related to this pathology, associated with rapid treatment initiation, improves survival, reducing mortality. This study opens the door for similar studies in other populations with a high rate of inbreeding. Our variants found in the study were different from those previously reported in other areas of Colombia, and it should be noted that we were able to diagnose all the symptomatic patients. This shows that in our population we can have our own variants that can be distributed throughout the country.

## Supporting information

S1 Table Clinical, biochemistry, and genetics characteristics of patients with HAE. (XLSX)
